# Role of Tumor Microenvironment in Prostate Cancer Immunometabolism

**DOI:** 10.3390/biom15060826

**Published:** 2025-06-06

**Authors:** Yutao Wang, Yiming Chen, Jianfeng Wang

**Affiliations:** 1 Department of Urology, Peking Union Medical College Hospital, Chinese Academy of Medical Sciences and Peking Union Medical College, Beijing 100005, China; ytwang96@cmu.edu.cn; 2 Department of Urology, The First Hospital of China Medical University, Shenyang 110001, China; chenyimingdr@163.com; 3 Institute of Urology, China Medical University, Shenyang 110001, China

**Keywords:** immunometabolism, metabolic reprogramming, prostate cancer, oxidative phosphorylation, tumor microenvironment

## Abstract

The tumor microenvironment (TME) plays a pivotal role in shaping immunometabolism in prostate cancer, influencing disease progression and therapeutic response. This review examines the dynamic interactions between tumor cells and immune cells within the prostate cancer TME, focusing on how metabolic reprogramming of both tumor and immune cells drives immunosuppression. Key immune players, including T-cells, macrophages, and myeloid-derived suppressor cells, undergo metabolic adaptations influenced by hypoxia, nutrient deprivation, and signaling from tumor cells. Additionally, we discuss the metabolic pathways involved, such as glycolysis and oxidative phosphorylation, and how these processes are exploited by cancer cells to evade immune surveillance. Furthermore, this review highlights potential therapeutic strategies targeting immunometabolism, including metabolic inhibitors and their combination with immunotherapies. A deeper understanding of the complex role of immunometabolism in prostate cancer will not only provide insights into the tumor’s immune evasion mechanisms but also facilitate the development of novel treatment approaches that enhance the efficacy of current therapies.

## 1. Introduction

Prostate cancer (PCa) is the second most commonly diagnosed cancer among men, contributing significantly to cancer mortality rates worldwide [1,2]. Based on the GLOBOCAN 2022 report, more than 1.4 million new prostate cancer cases were identified worldwide, with approximately 394,000 fatalities linked to the disease [3,4]. The incidence of prostate cancer varies based on geographic regions, with higher rates observed in developed regions, likely due to increased screening and the availability of diagnostic tools such as prostate-specific antigen (PSA) testing [5,6,7]. In contrast, lower detection rates in developing countries result in a higher prevalence of advanced-stage diagnoses, contributing to a greater mortality burden.

Prostate cancer is characterized by slow progression, with many cases remaining indolent and localized [8]. However, a subset of patients experience aggressive disease that metastasizes, most commonly to the bones, lymph nodes, liver, and lungs. The progression of prostate cancer is influenced by various factors, such as genetic predispositions, hormonal regulation, environmental influences, and lifestyle choices [9,10,11]. Signaling through androgen receptors (AR) is crucial for the normal maturation of the prostate gland as well as for the growth and advancement of prostate cancer. Consequently, androgen deprivation therapy (ADT) has become a fundamental treatment approach for advanced cases of the disease [12,13]. ADT reduces androgen levels or inhibits AR function, leading to tumor regression in many cases. However, resistance to ADT frequently occurs, leading to castration-resistant prostate cancer (CRPC), a more aggressive and lethal form of the disease [14,15]. Over the past decade, new therapeutic strategies have emerged for the management of prostate cancer. These include second-generation inhibitors of the androgen receptor pathway, such as enzalutamide and abiraterone, which provide further blockade of androgen signaling in CRPC patients [16,17]. Furthermore, the approval of immunotherapies like sipuleucel-T has added a novel dimension to treatment by leveraging the immune system to identify and eliminate prostate cancer cells. Despite these advancements, prostate cancer remains a major public health challenge, as many patients ultimately succumb to the disease due to resistance to treatments and metastasis.

Immunometabolism, a rapidly evolving field of cancer research, explores the intricate relationship between the immune system and metabolic pathways that drive immune cell function. Immunometabolism refers to the metabolic reprogramming of immune cells within the tumor microenvironment (TME) and its impact on their ability to mount effective antitumor responses [18,19,20]. Immune cells, including T-cells, macrophages, and dendritic cells, depend on specific metabolic pathways to support their activation, proliferation, and functional activities [21,22]. For instance, activated T-cells predominantly utilize glycolysis to meet their energy demands, whereas quiescent memory T-cells rely on oxidative phosphorylation (OXPHOS) for long-term survival [23,24].

In the TME, cancer cells compete with immune cells for scarce resources such as glucose, oxygen, and amino acids. This metabolic competition often favors the tumor cells, which undergo metabolic reprogramming to sustain their rapid proliferation and survival in adverse conditions, including hypoxia and nutrient scarcity [25,26]. Cancer cells predominantly rely on aerobic glycolysis, also known as the Warburg effect, to generate energy, even in the presence of sufficient oxygen [27,28]. This metabolic shift not only promotes the proliferation of cancer cells but also establishes an immunosuppressive environment by depleting vital nutrients in the TME and generating metabolic byproducts, such as lactate, which further suppress the antitumor immune response [29,30]. For example, T-cells that are deprived of glucose or subjected to high levels of lactate are less effective in mounting a cytotoxic response against cancer cells. Similarly, tumor-associated macrophages (TAMs) often undergo a metabolic shift towards fatty acid oxidation (FAO) and OXPHOS, which promotes their polarization towards an immunosuppressive M2 phenotype, supporting tumor growth and progression [31,32]. By understanding the metabolic changes that occur within the TME, researchers can identify novel therapeutic targets aimed at reprogramming the immune response to more effectively combat cancer.

The TME comprises the intricate and dynamic network of non-cancerous cells and extracellular components that surround and interact with tumor cells. In prostate cancer, the TME consists of various cellular elements, including immune cells, stromal cells (fibroblasts and mesenchymal stem cells), endothelial cells, and the extracellular matrix (ECM) [33,34,35]. These components form a supportive niche that facilitates tumor growth, metastasis, and immune evasion. On one hand, immune cells can identify and eliminate cancer cells through their cytotoxic functions. For example, CD8+ cytotoxic T-cells are tasked with recognizing and eliminating tumor cells that present abnormal or mutated antigens [36,37]. On the other hand, the chronic inflammation associated with cancer can lead to an immunosuppressive environment that promotes tumor growth. TAMs, for example, can polarize into M2 macrophages, thereby facilitating immune evasion, angiogenesis, and tumor proliferation [37,38].

Alongside immune cells, the TME contains stromal cells, such as cancer-associated fibroblasts (CAFs). CAFs produce growth factors, cytokines, and matrix metalloproteinases (MMPs) that alter the ECM and form a physical barrier, hindering immune cell infiltration into the tumor. This remodeling also aids tumor cell invasion and metastasis by breaking down the ECM and releasing bound growth factors. The vasculature within the TME is often abnormal, characterized by disorganized and leaky blood vessels that contribute to hypoxia and nutrient deprivation. Hypoxia, in turn, triggers the activation of hypoxia-inducible factors (HIFs), which enhance angiogenesis, induce metabolic reprogramming, and facilitate immunosuppression [39,40]. The metabolic landscape of the TME is another critical factor in tumor progression. As previously noted, cancer cells engage in metabolic reprogramming to facilitate their rapid growth, often to the detriment of immune cells. The elevated glycolysis rate in cancer cells results in lactate accumulation, which acidifies the TME and impairs the activity of cytotoxic T-cells and NK cells [41]. Additionally, cancer cells deplete substantial amounts of glucose, glutamine, and other nutrients, leaving immune cells in a state of metabolic deprivation [42]. This competition for resources suppresses the immune response, enabling the tumor to escape immune detection and proliferate uncontrollably.

The purpose of this review is to explore the pivotal role of the tumor microenvironment in shaping immunometabolism in prostate cancer. By concentrating on the interactions among tumor cells, immune cells, and stromal components within the TME, this review aims to provide insights into how metabolic reprogramming within the TME contributes to immune evasion and tumor progression. In particular, we will explore the various ways in which prostate cancer alters the metabolic landscape of the TME. This review will also discuss potential therapeutic strategies that target immunometabolism within the TME, with the goal of enhancing immune cell function and improving treatment outcomes in prostate cancer patients. Gaining insights into the interaction between the tumor microenvironment and immunometabolism will illuminate the mechanisms of immune evasion and facilitate the development of novel therapies that disrupt these processes and restore effective antitumor immunity.

## 2. The Tumor Microenvironment in Prostate Cancer

The TME in prostate cancer is crucial for influencing tumor behavior, affecting cancer cell survival, proliferation, and metastasis. It consists of a complex network of diverse non-cancerous cells, the ECM, blood vessels, and signaling molecules, all of which dynamically interact with cancer cells to either promote or inhibit tumor progression [43]. Key components within the prostate cancer TME include CAFs, immune cells, endothelial cells, and the ECM [44].

CAFs are among the most prevalent stromal components in the prostate cancer TME [45,46]. CAFs arise from resident fibroblasts, mesenchymal stem cells, or epithelial cells that undergo epithelial-to-mesenchymal transition (EMT) [47,48]. These fibroblasts are instrumental in the progression of prostate cancer by secreting a range of cytokines, growth factors and MMPs [49,50,51,52,53]. These secreted factors promote ECM remodeling, angiogenesis, and immune suppression, creating a tumor-promoting microenvironment. In particular, CAFs enhance cancer cell invasion and metastasis by facilitating the breakdown of the ECM, allowing cancer cells to spread to distant organs. Additionally, they can influence the metabolism of both cancer cells and immune cells by modulating the availability of nutrients within the TME, further contributing to tumor progression and immune evasion [54,55].

The prostate cancer TME houses a diverse assortment of immune cells, such as T-cells, macrophages, dendritic cells, and myeloid-derived suppressor cells (MDSCs) [56,57,58]. T-cells are key players in the immune response to cancer. However, in the prostate cancer TME, T-cell functionality is frequently compromised due to the immunosuppressive nature of the environment. Prostate tumors are typically characterized by low levels of cytotoxic T-cell (CD8+ T-cell) infiltration and an increased presence of regulatory T-cells (Tregs), which contribute to tumor immune escape through the secretion of immunosuppressive cytokines [59,60]. In addition to T-cell dysregulation, tumor-associated macrophages (TAMs) are typically polarized towards an M2 phenotype in prostate cancer. These M2-like macrophages promote tissue repair and tumor progression by secreting anti-inflammatory cytokines, enhancing angiogenesis, and suppressing cytotoxic immune responses, whereas M1 macrophages with tumoricidal functions are markedly depleted [61]. Dendritic cells (DCs) are essential antigen-presenting cells that play a crucial role in initiating and regulating immune responses [62,63]. In prostate cancer, however, DCs frequently exhibit impaired function, resulting in insufficient T-cell activation and an ineffective antitumor immune response. MDSCs are immunosuppressive cells that are abundant in the prostate cancer TME. MDSCs inhibit T-cell activity and promote tumor progression by releasing immunosuppressive factors like arginase-1 and nitric oxide synthase [56,64].

Endothelial cells line blood vessels and are vital for delivering nutrients and oxygen to tumor cells [65,66]. In prostate cancer, the vasculature within the TME is often abnormal, characterized by disorganized and leaky blood vessels. This aberrant vasculature contributes to hypoxia (low oxygen levels), which is a hallmark of many solid tumors, including prostate cancer. Endothelial cells within the TME also play a role in immune regulation [67,68]. They can express immune checkpoint molecules such as programmed death-ligand 1 (PD-L1), which inhibits T-cell activity and aids immune evasion [69]. Moreover, endothelial cells can form a physical barrier that restricts immune cell infiltration into the tumor, further reducing the effectiveness of the immune response [70].

The ECM is a vital structural component of the prostate cancer TME, providing physical support for cells and influencing cell behavior through biochemical and mechanical signals [71,72]. The ECM in prostate cancer is often remodeled by enzymes such as MMPs, which degrade ECM proteins and facilitate cancer cell invasion and metastasis [52,73]. The stiffness and composition of the ECM can influence cancer cell behavior, including proliferation, migration, and resistance to therapy [74,75].

The immune landscape of the prostate cancer TME is highly dynamic and consists of various immune cell types that can either promote or inhibit tumor progression. The balance between pro-tumor and antitumor immune cells is essential in shaping the overall immune response within the TME (Table 1). CD4+ helper T-cells play a central role in orchestrating the immune response [76,77]. They can differentiate into various subsets, including Th1, Th2, and Th17 cells, depending on the cytokine signals they receive within the TME. Th1 cells promote antitumor immunity by producing interferon-gamma (IFN-γ), which activates macrophages and enhances CD8+ T-cell function. However, in the prostate cancer TME, CD4+ T-cells are often skewed towards Th2 and Th17 phenotypes, which promote immunosuppression and tumor progression [78,79].

CD8+ T-cells are the main effector cells that target and eliminate cancer cells. They identify tumor antigens displayed by major histocompatibility complex (MHC) class I molecules on cancer cells and trigger apoptosis by releasing perforin and granzyme. In prostate cancer, however, CD8+ T-cell activity is frequently inhibited by immunosuppressive cells such as Tregs and MDSCs, as well as the expression of immune checkpoint molecules (e.g., PD-1/PD-L1) within the TME [36,60]. Tregs are a subset of CD4+ T-cells that are crucial for maintaining immune balance by suppressing excessive immune responses. In the prostate cancer TME, Tregs are often recruited in large numbers, where they inhibit the activity of CD8+ cytotoxic T-cells and other effector immune cells, thereby fostering an immunosuppressive environment that enables tumor cells to escape immune elimination.

Macrophages are highly plastic immune cells that can adopt either a pro-inflammatory (M1) or anti-inflammatory (M2) phenotype based on the signals they receive from the TME. In prostate cancer, TAMs are typically polarized towards the M2 phenotype, which is associated with tumor promotion. Additionally, M2 macrophages promote angiogenesis and tissue remodeling by secreting vascular endothelial growth factor (VEGF) and MMPs. MDSCs represent a diverse group of immature myeloid cells that accumulate in the TME as the tumor progresses [56,57]. These cells have potent immunosuppressive properties; they inhibit the activation and function of T-cells and NK cells by producing reactive oxygen species (ROS), nitric oxide (NO) and arginase. MDSCs also promote the expansion of Tregs, further enhancing the immunosuppressive environment in the prostate cancer TME. The presence of MDSCs is linked to a poor prognosis in prostate cancer patients, as they facilitate tumor immune evasion and resistance to immunotherapy. NK cells are innate immune cells that play a critical role in recognizing and eliminating tumor cells that have downregulated MHC class I molecules [80,81]. However, in the prostate cancer TME, NK cell function is often impaired due to the presence of immunosuppressive factors. As a result, NK cells are less effective at controlling tumor progression in prostate cancer.

The interaction between tumor cells and immune cells within the prostate cancer TME is a key factor in shaping the immune response and influencing tumor progression. Tumor cells secrete a variety of factors that regulate immune cell activity, leading to immunosuppression and tumor escape [82]. One of the main mechanisms by which tumor cells evade immune elimination is the expression of immune checkpoint molecules, such as PD-L1. When PD-L1 on tumor cells binds to PD-1 on T-cells, inhibitory signals are transmitted that suppress T-cell activation and function. This allows tumor cells to evade immune surveillance and proliferate uncontrollably. The overexpression of immune checkpoint molecules in the prostate cancer TME has led to the development of immune checkpoint inhibitors (ICIs), such as anti-PD-1 and anti-CTLA-4 therapies, which aim to restore T-cell activity and promote tumor regression.

Prostate cancer cells secrete a variety of immunosuppressive cytokines, including TGF-β, IL-10 and prostaglandin E2 (PGE2), which inhibit the activation of effector immune cells and promote the expansion of Tregs and MDSCs [83,84]. These cytokines create an immunosuppressive microenvironment that limits the ability of the immune system to mount an effective antitumor response. As mentioned earlier, tumor cells and immune cells compete for nutrients within the TME. Tumor cells, which rely heavily on aerobic glycolysis, consume large amounts of glucose, depriving immune cells of the energy they need to function. The accumulation of metabolic byproducts, such as lactate, further inhibits immune cell function by creating an acidic environment that impairs T-cell and NK cell activity.

**Table 1:** Immune Cell Types And Their Functions In the Prostate Cancer TME.

Immune Cell Type	Function	Mechanisms of Immunosuppression
CD4+ T-cells	Coordinate immune response; differentiate into Th1, Th2, and Th17 cells; promote or inhibit tumor immunity.	Skewing towards Th2 and Th17 phenotypes; produce pro-inflammatory cytokines like IL-4 and IL-17 [85].
CD8+ T-cells	Primary effector cells for killing cancer cells; recognize tumor antigens; induce apoptosis.	Suppressed by immunosuppressive cells and checkpoint molecules (e.g., PD-1/PD-L1) [86].
Regulatory T-cells (Tregs)	Maintain immune homeostasis; suppress excessive immune responses; inhibit CD8+ T-cell activity.	Inhibit CD8+ cytotoxic T-cell function and promote immunosuppression.
Tumor-Associated Macrophages (TAMs)	Highly plastic; can be M1 (pro-inflammatory) or M2 (anti-inflammatory); promote tumor progression in M2 phenotype [61].	Produce anti-inflammatory cytokines (IL-10, TGF-β) that suppress T-cells and promote angiogenesis [87].
MDSCs	Heterogeneous population that inhibits T-cell and NK cell activation; promote expansion of Tregs.	Produce ROS, NO, and arginase to inhibit immune cell functions; associated with poor prognosis [64].
Natural Killer (NK) cells	Recognize and eliminate tumor cells; impaired function due to immunosuppressive factors in the TME.	Impaired by factors like TGF-β and IL-10; less effective in controlling tumor growth [88,89].

## 3. Metabolic Reprogramming in Prostate Cancer

### 3.1. Overview of Metabolic Pathways

Metabolic reprogramming is a hallmark of cancer, including prostate cancer, where cells undergo significant changes in their energy production and biosynthetic pathways to meet the increased demands for rapid proliferation and survival. In normal cells, energy is primarily generated through OXPHOS in the mitochondria, a process that uses oxygen to produce adenosine triphosphate (ATP). However, cancer cells, including those in prostate cancer, frequently switch to aerobic glycolysis [90]. This shift from OXPHOS to glycolysis allows cancer cells to rapidly generate ATP and biosynthetic intermediates required for the synthesis of nucleotides, lipids and proteins, supporting their accelerated proliferation.

Key metabolic pathways altered in cancer include glycolysis, OXPHOS, FAO and glutaminolysis [91,92]. Glycolysis is a process that breaks down glucose into pyruvate, producing ATP and lactate [93,94]. In prostate cancer cells, glycolysis becomes the dominant metabolic pathway, providing not only energy but also intermediates for anabolic processes, such as lipid and nucleotide synthesis, essential for sustaining rapid cell division. This metabolic reprogramming is driven by oncogenes and tumor suppressor gene mutations.

OXPHOS, which occurs in the mitochondria, remains an important pathway in cancer cells but is often downregulated in favor of glycolysis. OXPHOS is less efficient in terms of speed, but it yields a higher amount of ATP per glucose molecule than glycolysis [95,96]. Some prostate cancer cells maintain functional OXPHOS, particularly in the early stages of tumorigenesis or when oxygen levels are sufficient. However, as the tumor grows and hypoxic conditions develop, the reliance on glycolysis increases. FAO is another critical metabolic pathway in prostate cancer. Cancer cells use FAO to generate ATP and produce lipid precursors for membrane biosynthesis. FAO is particularly relevant in prostate cancer, where lipid metabolism is highly active, especially in advanced CRPC [31,32]. The increased reliance on lipid metabolism helps cancer cells survive in nutrient-poor and hypoxic environments by providing an alternative source of energy. Glutaminolysis, the metabolic pathway that breaks down glutamine to provide energy and carbon skeletons for biosynthesis, is also upregulated in prostate cancer. Glutamine is an important substrate for generating α-ketoglutarate, an intermediate in the tricarboxylic acid (TCA) cycle, which supports the anabolic processes in rapidly dividing cancer cells [97,98]. By increasing the uptake and utilization of glutamine, prostate cancer cells can fuel their proliferation and maintain redox balance, ensuring their survival under stressful conditions.

### 3.2. Metabolic Adaptations of Tumor Cells in Prostate Cancer

Prostate cancer cells exhibit metabolic plasticity, allowing them to adapt to the dynamic and often hostile conditions of the TME. This reprogramming enables the tumor cells to not only support their rapid proliferation but also to evade immune detection and therapy. One of the primary ways prostate cancer cells achieve this is through the upregulation of glycolysis [90]. The Warburg effect, a hallmark of cancer metabolism, plays a significant role in prostate cancer. By favoring glycolysis over OXPHOS, prostate cancer cells can produce ATP more quickly, albeit less efficiently [99,100]. However, the byproducts of glycolysis, such as lactate, are not simply waste products; they contribute to shaping the TME by promoting acidosis, which suppresses the function of immune cells and enhances cancer cell invasiveness [101]. In addition to glycolysis, prostate cancer cells also activate alternative metabolic pathways, such as FAO and glutaminolysis, to ensure their survival in nutrient-depleted and hypoxic environments [102,103]. These pathways provide essential building blocks for lipid and protein synthesis, which are crucial for cell proliferation and division.

**Figure 1:**
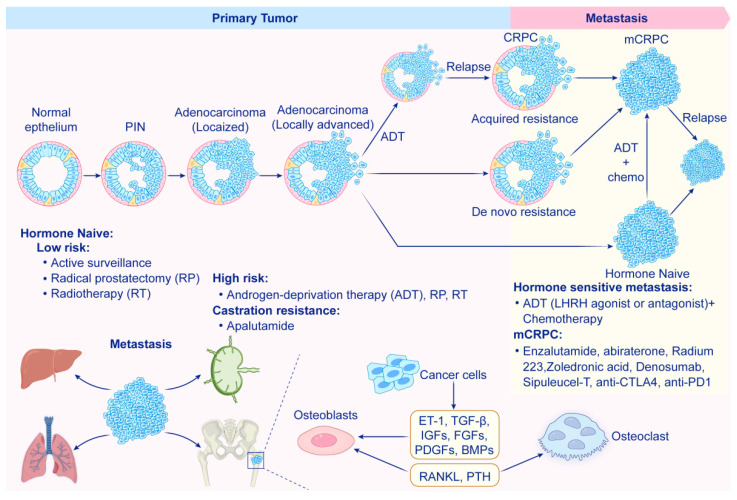
The progression of prostate cancer leads to the development of metastatic castration-resistant prostate cancer (mCRPC). The diagnosis of prostatic intraepithelial neoplasia (PIN) is characterized by the proliferation of luminal cells alongside dysplasia within the ducts. PIN has the potential to progress to localized prostate adenocarcinoma, which can then develop into locally invasive carcinoma when the basal cell layer is compromised, allowing cancer cells to invade the basal lamina. Once locally advanced, prostate cancer typically metastasizes first to the draining lymph nodes and later to distant sites, primarily the bones, liver and lungs, with bone being the most common metastatic site. In cases of bone metastasis, there is a dynamic interaction among cancer cells, osteoblasts and osteoclasts, creating a “vicious cycle” of bone formation and destruction that ultimately promotes cancer cell survival and tumor progression. Androgen receptor (AR)-dependent localized advanced prostate adenocarcinoma initially responds to androgen deprivation therapy (ADT) but may progress to castration-resistant prostate cancer (CRPC). Some localized advanced cases can show de novo resistance to ADT. Similarly, AR-dependent hormone-naïve metastatic tumors often respond to ADT at first but can evolve into metastatic CRPC, while AR-indifferent hormone-naïve metastatic tumors may exhibit de novo resistance. Treatment strategies for prostate cancer are guided by the tumor stage and the patient’s prior therapies, requiring a personalized approach to management as the disease progresses.

Another critical factor in the metabolic reprogramming of prostate cancer cells is androgen receptor (AR) signaling. The AR is fundamental to the biology of prostate cancer, regulating the expression of genes associated with cell proliferation, differentiation and survival. In prostate cancer, AR signaling drives the metabolic reprogramming of tumor cells by enhancing the expression of genes involved in glycolysis, lipid synthesis and mitochondrial function [104,105]. For example, the activation of the AR enhances the expression of enzymes such as hexokinase 2 (HK2) and pyruvate kinase M2 (PKM2), which play essential roles in regulating glycolysis [106,107]. Additionally, AR signaling facilitates lipid metabolism by increasing the expression of genes associated with fatty acid synthesis, including fatty acid synthase (FASN) [108]. This metabolic shift supports the energy demands of rapidly proliferating prostate cancer cells and contributes to resistance to therapy, particularly ADT. In advanced prostate cancer, particularly CRPC, metabolic reprogramming becomes even more pronounced (Figure 1). Tumor-derived exosomes play a pivotal role in this process. These nanoscale vesicles remodel metabolic pathways between cancer cells and stromal components by transferring bioactive molecules, thereby driving tumor progression [109,110]. For example, exosomes facilitate CRPC cells’ adaptation to low-androgen conditions by enhancing glycolysis, promoting fatty acid synthesis and altering glutamine utilization [102,111]. Simultaneously, they induce metabolic reprogramming in stromal cells such as fibroblasts, endothelial cells and immune cells, contributing to the establishment of a tumor-promoting microenvironment [112]. These adaptations allow CRPC cells to survive despite AR inhibition, underscoring the significance of targeting metabolic pathways as a therapeutic approach for this aggressive cancer variant.

### 3.3. Impact of Tumor Metabolism on the Immune Microenvironment

**Figure 2:**
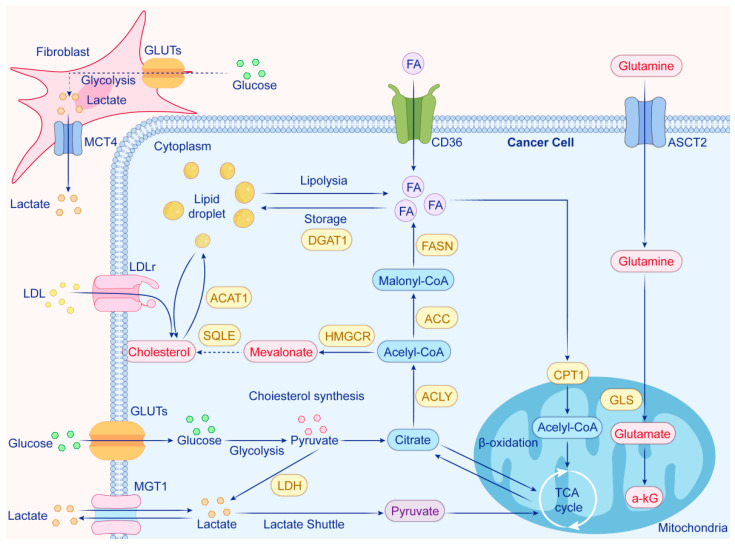
In advanced prostate cancer (PCa), cancer cells undergo significant metabolic changes to meet their increased energy and proliferation demands. Key features include the following: 1. Increased Glucose Uptake: PCa cells overexpress glucose transporters (GLUTs), leading to enhanced glycolysis and lactate production via lactate dehydrogenase (LDH). Lactate is exported through monocarboxylate transporters (MCTs) and can be taken up again by cancer cells to fuel the tricarboxylic acid (TCA) cycle. 2. Glutamine Utilization: Glutamine is transported into cells via ASCT2, converted to glutamate by glutaminase (GLS), and then transformed into α-ketoglutarate, entering the TCA cycle. 3. Fatty Acid Metabolism: Fatty acids are taken up through the CD36 transporter and undergo β-oxidation in mitochondria to produce acetyl-CoA, which fuels the TCA cycle. Excess fatty acids can be stored as lipid droplets or undergo lipolysis. 4. Cholesterol Biosynthesis: Cholesterol is synthesized from acetyl-CoA via the mevalonate pathway, involving enzymes such as HMGCR and SQLE, and can be stored in lipid droplets or internalized from low-density lipoprotein (LDL). This metabolic reprogramming enables PCa cells to adapt and thrive, highlighting potential therapeutic targets in these pathways.

The metabolic reprogramming of prostate cancer cells has profound effects on the immune microenvironment, as the altered metabolism of tumor cells competes with and influences the function of immune cells within the TME (Figure 2). Prostate cancer cells utilize significant quantities of glucose via aerobic glycolysis, depriving immune cells such as T-cells of the glucose necessary for their activation and function. This competition for nutrients results in a shortage for immune cells, impairing their capacity to mount an effective antitumor response [113,114]. CD8+ T-cells rely on glycolysis for energy during their activation and effector phases. However, in the nutrient-poor TME, T-cells are often unable to obtain sufficient glucose, leading to their dysfunction and exhaustion. In addition to nutrient competition, prostate cancer cells secrete metabolic byproducts, such as lactate, that further suppress immune function [115,116]. Lactate, produced in large quantities through glycolysis, contributes to the acidification of the TME, which creates a hostile environment for immune cells. Lactate also promotes the polarization of macrophages towards an immunosuppressive M2 phenotype, which supports tumor progression and metastasis [117]. In this way, the metabolic reprogramming of prostate cancer cells not only sustains their survival but also actively suppresses the immune response, allowing the tumor to evade immune detection.

Moreover, prostate cancer cells produce other immunosuppressive metabolites, such as adenosine, which accumulates in the TME and exerts potent immunosuppressive effects [61]. Adenosine binds to its receptors on immune cells, particularly T-cells and NK cells, inhibiting their activation and reducing their ability to eliminate tumor cells [118]. The accumulation of adenosine in the TME is often a consequence of hypoxia, which induces the expression of enzymes such as CD39 and CD73 that convert ATP to adenosine [119]. In addition to lactate and adenosine, prostate cancer cells also alter immune cell metabolism by producing ROS and depleting essential amino acids such as tryptophan [120]. The generation of ROS in the TME leads to oxidative stress, which impairs T-cell receptor signaling and reduces the cytotoxic activity of CD8+ T-cells. Similarly, the depletion of tryptophan by the enzyme indoleamine 2,3-dioxygenase (IDO), which is produced by prostate cancer cells, results in the inhibition of T-cell proliferation and activation [121]. This metabolic manipulation of the immune microenvironment by prostate cancer cells creates a highly immunosuppressive TME that is resistant to immune-mediated tumor eradication.

## 4. Immunometabolic Changes in the Tumor Microenvironment

The TME plays a critical role in shaping the metabolic behavior of immune cells, influencing their ability to mount an effective antitumor response. In prostate cancer, the TME is characterized by hypoxia, nutrient deprivation and accumulation of metabolic byproducts, all of which contribute to a hostile environment that suppresses immune cell function [122]. Immunometabolism, the study of how metabolic processes regulate immune cell activity, has become a key area of interest in understanding how prostate cancer manipulates the immune response. The metabolic alterations in immune cells within the TME are driven by both the intrinsic metabolic demands of the tumor and the external pressures of the TME.

### 4.1. T-Cell Metabolism in the TME

The activation and function of T-cells are largely dependent on their metabolic state, which is tightly regulated by the availability of nutrients and oxygen in the TME. Glycolysis allows T-cells to generate energy rapidly to support the increased demands of proliferation and cytokine production during an immune response. However, this metabolic switch is highly sensitive to the conditions of the TME.

In the prostate cancer TME, tumor cells utilize substantial amounts of glucose via aerobic glycolysis, commonly known as the Warburg effect, depriving immune cells such as T-cells of essential glucose [123,124]. This creates a nutrient-deprived environment in which T-cells are unable to undergo the glycolytic switch necessary for full activation. Instead, T-cells are forced to rely on OXPHOS, a more efficient but slower process that does not meet the energy demands required for rapid immune responses. As a result, T-cells become metabolically exhausted, impairing their ability to produce pro-inflammatory cytokines and eliminate tumor cells. This exhaustion is particularly evident in CD8+ cytotoxic T-cells, which are essential for directly attacking and eliminating cancer cells. In addition to glucose deprivation, the accumulation of metabolic byproducts, such as lactate, further inhibits T-cell function.

Another key factor that influences T-cell metabolism in the prostate cancer TME is hypoxia. As tumors grow, the oxygen demand often exceeds the supply, leading to areas of low oxygen tension within the TME. HIFs, particularly HIF-1α, are activated in response to low oxygen levels [125,126]. In T-cells, HIF-1α promotes the shift towards glycolysis under hypoxic conditions. However, in the prostate cancer TME, where glucose is limited, T-cells are unable to fully activate glycolysis, leading to dysfunction and immune suppression. Additionally, hypoxia enhances the expression of immune checkpoint molecules, such as PD-L1, on both tumor and immune cells, further reducing T-cell activity. This metabolic reprogramming of T-cells in response to the prostate cancer TME is a key mechanism by which the tumor evades immune surveillance and proliferates.

### 4.2. Macrophage Polarization and Metabolism

Macrophages are highly plastic immune cells that can adopt different functional states depending on the signals they receive from the TME. The metabolic profiles of these phenotypes are distinct and play a critical role in determining their function within the TME. M1 macrophages are associated with antitumor immunity and are characterized by a high level of glycolytic activity. Glycolysis provides the necessary energy for the production of proinflammatory cytokines, such as tumor necrosis factor-alpha (TNF-α) and interleukin-1β (IL-1β), which help activate other immune cells and promote tumor cell elimination. In contrast, M2 macrophages, which support tumor progression and metastasis, rely more heavily on OXPHOS and FAO for their energy needs [127,128].

Within the prostate cancer TME, tumor cells release a range of cytokines and growth factors, such as colony-stimulating factor 1 (CSF-1) and IL-4, which promote the polarization of macrophages toward the M2 phenotype [129,130]. The metabolic environment of the TME further supports this polarization. For example, the hypoxic conditions commonly found in the prostate cancer TME promote the activation of HIF-1α in macrophages, which induces a switch from glycolysis to OXPHOS and FAO, favoring the M2 phenotype [131]. Additionally, the presence of metabolic byproducts, such as lactate, also promotes M2 polarization [117] The metabolic reprogramming of macrophages in the prostate cancer TME contributes to the creation of an immunosuppressive environment that supports tumor progression and metastasis. M2 macrophages not only suppress the activity of cytotoxic T-cells but also promote the recruitment of other immunosuppressive cells, such as MDSCs and regulatory T-cells (Tregs), further enhancing immune evasion [132].

### 4.3. MDSCs and Immunosuppression

MDSCs are a diverse group of immature myeloid cells that accumulate in the TME as the tumor progresses. MDSCs are potent immunosuppressive cells that inhibit the function of both T-cells and NK cells, allowing tumors to evade immune surveillance. The expansion and recruitment of MDSCs in the TME are driven by a variety of factors, including metabolic changes in both tumor cells and immune cells. MDSCs are highly dependent on glycolysis for their energy needs, and this reliance on glycolysis is a key feature of their immunosuppressive function [133]. In the nutrient-deprived environment of the prostate cancer TME, MDSCs outcompete other immune cells for glucose, using it to fuel their own metabolic needs. This not only deprives T-cells of the glucose required for their activation but also allows MDSCs to produce large amounts of lactate and ROS, both of which contribute to the suppression of T-cell function [92,134].

The hypoxic conditions of the TME also play a critical role in the metabolic reprogramming of MDSCs. Hypoxia activates HIF-1α in MDSCs, which promotes glycolysis and enhances their immunosuppressive activity. Furthermore, hypoxia induces the expression of enzymes [121,135]. Tryptophan depletion results in the suppression of T-cell proliferation and activation, further amplifying the immunosuppressive effects of MDSCs. Beyond their impact on T-cells, MDSCs also facilitate the expansion of other immunosuppressive cell types, such as Tregs, and release cytokines such as IL-10 and TGF-β, which inhibit the activity of effector immune cells [133]. By establishing a highly immunosuppressive microenvironment, MDSCs are crucial in enabling prostate cancer to evade immune elimination and persist in its proliferation.

### 4.4. Other Immune Cells Affected by TME Metabolism

In addition to T-cells, macrophages and MDSCs, other immune cells in the prostate cancer TME, such as DCs, NK cells and regulatory T-cells (Tregs), are also affected by the altered metabolic landscape of the tumor. DCs often exhibit impaired function due to metabolic constraints in the prostate cancer TME [136]. Like T-cells, DCs require glucose for glycolysis to support their activation and ability to present antigens to T-cells. In the nutrient-deprived TME, DCs are unable to access sufficient glucose, leading to their dysfunction. Moreover, the accumulation of lactate and other metabolic byproducts in the TME inhibits the maturation of dendritic cells, preventing them from effectively activating T-cells [137]. Consequently, the antitumor immune response is compromised, enabling prostate cancer cells to evade detection and continue proliferating.

Natural killer (NK) cells are responsible for recognizing and eliminating tumor cells that have downregulated MHC class I molecules, a common feature of cancer cells [138]. Like T-cells, NK cells rely on glycolysis for energy during their activation and cytotoxic activity. In the glucose-deprived environment of the prostate cancer TME, NK cells are unable to sustain the metabolic demands of their cytotoxic function, leading to reduced tumor-killing activity. Moreover, the presence of immunosuppressive cytokines, such as TGF-β, further inhibits NK cell function by promoting a metabolic shift away from glycolysis and towards OXPHOS, impairing their ability to respond to tumor cells [139]. In the prostate cancer TME, Tregs are often recruited in large numbers, where they suppress the activity of cytotoxic T-cells and other effector immune cells. Tregs have a distinct metabolic profile compared to other T-cell subsets, relying more heavily on OXPHOS and FAO for their energy needs [140]. This metabolic flexibility allows Tregs to thrive in the nutrient-deprived and hypoxic conditions of the TME, where other immune cells struggle to survive.

## 5. Hypoxia and Immunometabolism in Prostate Cancer

### 5.1. Role of Hypoxia in Prostate Cancer Progression

Hypoxia, or decreased oxygen availability, is a common characteristic of solid tumors, including prostate cancer. As prostate tumors grow, their increasing size and rapid proliferation outstrip the supply of oxygen delivered through the existing vasculature. The abnormal, disorganized and leaky blood vessels in the TME further exacerbate this oxygen shortage, leading to areas of chronic hypoxia [141,142]. These hypoxic zones are not merely a byproduct of tumor progression but actively contribute to cancer progression by driving aggressive behavior in prostate cancer cells Under hypoxic conditions, cancer cells must adapt to survive. Prostate cancer cells activate various signaling pathways that enable them to continue proliferating, invading and surrounding tissues despite the lack of oxygen. Hypoxia promotes tumor aggressiveness by inducing changes that increase the cells’ metastatic potential, enhance their resistance to therapy and stimulate angiogenesis to restore oxygen supply. One of the primary pathways activated by hypoxia is the HIF pathway, which is essential for regulating the cellular response to low oxygen levels [143,144].

In prostate cancer, hypoxia is linked to poor prognosis, a higher likelihood of metastasis, and resistance to treatments such as radiation therapy and chemotherapy. The hypoxic environment fosters a more invasive phenotype, enabling cancer cells to migrate and invade distant tissues, contributing to the development of metastatic CRPC. Hypoxia-induced alterations in prostate cancer cells not only enhance their survival but also foster the development of an immunosuppressive microenvironment that allows the tumor to evade immune detection and elimination.

### 5.2. Hypoxia-Induced Metabolic Shifts

Hypoxia profoundly alters the metabolism of prostate cancer cells, forcing them to adopt alternative metabolic pathways to generate energy and biosynthetic precursors [145]. In oxygen-rich conditions, cells rely primarily on OXPHOS to produce ATP in the mitochondria. OXPHOS is a highly efficient process that requires oxygen to drive the electron transport chain, producing large amounts of ATP. However, in the oxygen-deprived environment of the prostate cancer TME, cells are unable to maintain OXPHOS and must switch to less efficient metabolic pathways to survive.

One of the most well-known metabolic adaptations to hypoxia is the Warburg effect, whereby cancer cells shift from OXPHOS to aerobic glycolysis, even when oxygen is present [146]. Under hypoxic conditions, this shift is more pronounced because glycolysis does not require oxygen and can proceed under low oxygen tension. Glycolysis produces ATP at a much faster rate than OXPHOS, albeit less efficiently in terms of ATP per glucose molecule [147]. Nevertheless, it allows cancer cells to rapidly generate the energy needed to sustain their proliferation and division under adverse conditions. In addition to generating ATP, glycolysis also produces intermediate molecules that are used for biosynthesis, enabling prostate cancer cells to synthesize the building blocks necessary for cell proliferation. The shift to glycolysis under hypoxia also results in the accumulation of lactate, a byproduct of anaerobic metabolism. Lactate accumulation contributes to the acidification of the TME, which not only supports cancer cell invasion but also creates an environment that impairs immune cell function, further promoting immune evasion [148,149]. Additionally, hypoxia activates enzymes involved in other metabolic pathways, such as FAO and glutaminolysis, which provide alternative sources of energy and carbon skeletons for anabolic processes [150,151]. These metabolic shifts allow prostate cancer cells to thrive in nutrient-deprived and oxygen-deprived environments, enhancing their survival and aggressive behavior.

### 5.3. Influence of Hypoxia on Immune Cells

Hypoxia not only changes the metabolism of prostate cancer cells but also has a profound effect on the metabolism and function of immune cells within the TME. Immune cells, such as T-cells, macrophages and dendritic cells, rely on specific metabolic pathways to facilitate their activation, proliferation and effector functions [152]. Hypoxia disrupts these processes by inducing metabolic adaptations that impair immune cell function and promote the development of an immunosuppressive microenvironment. One of the main ways hypoxia affects immune cells is through the activation of HIFs, specifically HIF-1α. HIFs are transcription factors that orchestrate cellular responses to hypoxic conditions by enhancing their stability and regulating the expression of genes involved in metabolic pathways [153]. In immune cells, HIF-1α promotes the conversion from OXPHOS to glycolysis, similar to its role in cancer cells. However, as previously mentioned, immune cells are often unable to maintain effective function under the glycolytic conditions of the TME, where glucose is scarce and lactate accumulates.

In T cells, beyond the hypoxia-induced competition for glucose, the accumulation of lactate leads to acidification of the TME, which further impairs T cell function by suppressing cytokine production and reducing the cytotoxic activity of CD8^+^ T cells against tumor cells [154]. Hypoxia also promotes the stabilization of HIF-1α, which drives the polarization of macrophages toward the M2 phenotype, characterized by immunosuppressive and pro-tumoral functions, rather than the pro-inflammatory and antitumor M1 phenotype [127]. In DCs, hypoxia compromises their maturation and antigen-presenting capabilities, thereby attenuating the initiation of adaptive immune responses [155]. Collectively, these hypoxia-mediated alterations facilitate immune evasion and tumor progression. As such, targeting hypoxia represents a promising therapeutic strategy to restore immune competence and enhance antitumor immunity in prostate cancer.

## 6. Targeting Immunometabolism in Prostate Cancer Therapy

Targeting immunometabolism in prostate cancer therapy is an emerging approach aimed at overcoming the immunosuppressive TME and enhancing the effectiveness of existing therapies. As research uncovers the intricate metabolic interactions between tumor cells and immune cells, therapeutic strategies that disrupt these metabolic processes are gaining attention for their potential to reinvigorate immune responses and inhibit tumor progression. This section discusses current therapeutic approaches that target the TME, explores metabolic inhibitors in prostate cancer therapy, examines combination therapies, and looks toward future directions for immunometabolism-based treatments in prostate cancer.

### 6.1. Current Therapeutic Approaches Targeting the TME

Immunotherapies that leverage the immune system to combat cancer have significantly transformed treatment approaches in recent years. Although these therapies have demonstrated efficacy in cancers such as melanoma and lung cancer, their success in prostate cancer has been limited. The immunosuppressive nature of the prostate cancer microenvironment, characterized by metabolic dysfunction and immune evasion, poses significant challenges to the effectiveness of these treatments. A key form of immunotherapy is ICIs, which target inhibitory molecules such as PD-1 and CTLA-4 that suppress immune responses [156,157]. By blocking these immune checkpoints, ICIs reinvigorate T-cells, enhancing their ability to recognize and eliminate cancer cells. However, given the low levels of T-cell infiltration and the abundance of MDSCs in the prostate cancer TME, combination approaches have become necessary. These strategies include co-administration of PD-1/PD-L1 inhibitors with CTLA-4 antagonists or integration with MDSC-depleting agents or tumor vaccines to overcome resistance and enhance antitumor immunity [61]. To date, many clinical trials have been performed to assess the therapeutic efficacy of immune checkpoint blockade on clinical outcomes in PCa patients (Table 2) [158].

CAR-T cell therapy is another promising immunotherapeutic approach that has revolutionized the treatment of certain hematological malignancies [159,160]. CAR-T cells are T-cells that have been genetically engineered to express receptors that specifically target tumor-associated antigens. In prostate cancer, researchers are exploring CAR-T cells targeting prostate-specific membrane antigen (PSMA), a protein frequently overexpressed on prostate cancer cells. While early results in solid tumors, including prostate cancer, have been less successful than in blood cancers, modifications in CAR-T cell engineering and delivery, including improvements in their metabolic function, are under investigation to improve their efficacy in solid tumors [161].

One of the reasons for the limited success of immunotherapies in prostate cancer is the hostile TME, where immune cells are metabolically suppressed by nutrient deprivation, hypoxia and the accumulation of metabolic byproducts like lactate [122]. Therefore, strategies to remodel the TME and reverse its immunosuppressive features have emerged as a key focus. Inhibition of the VEGF pathway, thereby counteracting hypoxia induced by aberrant angiogenesis, remains a cornerstone of TME-directed therapy [162]. Metabolic interventions, such as inhibition of lactate production or disruption of FAO, aim to restore immune cell metabolic function [163,164]. Moreover, the development of advanced drug delivery systems has significantly improved intratumoral penetration and payload delivery efficiency, thereby enhancing tumor cytotoxicity and augmenting antitumor immune activation [44]. These multimodal strategies hold promise for converting the TME from “cold” to “hot”, thereby improving therapeutic response rates and overall survival in PCa patients.

### 6.2. Metabolic Inhibitors in Cancer Therapy

Given the critical role of metabolic reprogramming in the prostate cancer TME, targeting specific metabolic pathways has emerged as a strategy to boost immune responses and impair tumor progression. One of the most well-studied targets is glycolysis, a process by which tumor cells generate energy and biosynthetic precursors under hypoxic conditions. By inhibiting glycolysis, researchers aim to reduce the availability of glucose in the TME, depriving tumor cells of a critical energy source while simultaneously restoring glucose levels for immune cells. Inhibitors of key glycolytic enzymes, such as 2-deoxyglucose (2-DG), have shown potential in preclinical models of prostate cancer by inhibiting tumor progression and enhancing T-cell function. However, glycolysis inhibitors pose challenges, as they may have off-target effects on normal cells that rely on glycolysis, necessitating a careful balance between efficacy and toxicity [28,165].

Another target is fatty acid metabolism, particularly FAO, a key metabolic pathway in both tumor cells and immunosuppressive macrophages within the TME. In prostate cancer, tumor cells often rely on FAO to generate ATP and promote survival under nutrient-deprived conditions [31]. Glutaminolysis, a process by which cells utilize glutamine as a key fuel source, is another metabolic pathway that is frequently upregulated in prostate cancer [166]. Tumor cells utilize glutamine to support the TCA cycle, supplying energy and biosynthetic intermediates. Targeting glutaminolysis with inhibitors like CB-839 (telaglenastat), a glutaminase inhibitor, has been shown to inhibit tumor progression in preclinical studies by limiting the availability of key substrates for tumor cell metabolism [167].

### 6.3. Combination Therapies

The complexity of the prostate cancer TME and its immunosuppressive metabolic environment has led to increasing interest in combination therapies that integrate metabolic inhibitors with existing immunotherapies. By combining these approaches, researchers aim to disrupt the metabolic constraints that impair immune cell function. Glycolysis inhibitors can reduce the competitive advantage of tumor cells in the TME, increasing glucose availability for immune cells, particularly T-cells [168]. This not only enhances T-cell function but also mitigates the immunosuppressive effects of lactate accumulation in the TME.

Similarly, combining FAO inhibitors with immunotherapies is another promising strategy. Pharmacological inhibition of FAO in TAMs promotes a phenotypic shift from the immunosuppressive M2 phenotype to a pro-inflammatory M1 phenotype, thereby enhancing the efficacy of ICIs by mitigating immunosuppressive signaling within the TME [169,170]. Preclinical studies have shown that FAO inhibitors can improve the efficacy of CAR-T- cell therapy by creating a more favorable metabolic environment for CAR-T cells to function in solid tumors such as prostate cancer [171]. Glutaminolysis inhibitors are also being explored in combination with ICIs and other immunotherapies. By reducing the availability of glutamine for tumor cells, glutaminolysis inhibitors can enhance the metabolic fitness of immune cells, particularly T-cells and dendritic cells, thereby improving their ability to mount an effective antitumor response [172]. Emerging evidence indicates that the concurrent application of glutaminase inhibitors with immune checkpoint blockade (e.g., PD-L1 or CTLA4) enhances T-cell infiltration within the TME and amplifies antitumor immune responses, thereby underscoring the therapeutic potential of these combination strategies [173,174]. In addition to these combinations, researchers are exploring the integration of metabolic inhibitors with other cancer therapies, such as radiation therapy and chemotherapy. Metabolic inhibitors may enhance the sensitivity of tumor cells to radiation and chemotherapy by disrupting their energy supply and reducing their ability to repair DNA damage.

### 6.4. Future Directions in Immunometabolism-Based Therapies

The area of immunometabolism-based therapies is still in its infancy, yet it shows considerable promise for enhancing treatment outcomes in prostate cancer. As our understanding of metabolic interactions within the TME deepens, new therapeutic strategies are emerging that could reprogram the metabolism of both tumor and immune cells to enhance antitumor immunity.

One promising direction for future research is the development of more selective metabolic inhibitors that specifically target tumor or immunosuppressive cells within the microenvironment, while minimizing impact on normal tissues. This approach would lower the risk of off-target effects and improve the therapeutic efficacy of these inhibitors. Additionally, advancements in precision medicine and biomarker development could identify patients who would benefit most from specific metabolic inhibitors, enabling more personalized prostate cancer treatment strategies.

Another area of interest is metabolic reprogramming as a therapeutic strategy. Rather than simply inhibiting metabolic pathways, researchers are exploring ways to reprogram the metabolism of immune cells to enhance their function in the TME [175,176]. For example, approaches that enhance the metabolic function of T-cells by improving their glycolytic or mitochondrial function could increase the efficacy of immunotherapies for prostate cancer.

Despite the promising potential of immunometabolism-based therapies, there are significant challenges to overcome. One of the major challenges is the heterogeneity of the prostate cancer TME, which can vary significantly between patients and even within different regions of the same tumor [176,177]. This heterogeneity makes it challenging to predict which metabolic pathways are most important in a given patient and complicates the development of effective therapies. Additionally, the metabolic demands of tumor cells and immune cells are highly dynamic, changing in response to factors such as nutrient availability, hypoxia and treatment. This dynamic nature of metabolism poses a challenge for designing therapies that can effectively target the metabolic vulnerabilities of prostate cancer over time.

In conclusion, targeting immunometabolism in prostate cancer therapy represents a promising strategy to enhance the effectiveness of existing treatments and overcome the challenges posed by the immunosuppressive TME. By disrupting the metabolic pathways that support tumor progression and suppress immune responses, metabolic inhibitors have the potential to reinvigorate the immune system and improve treatment outcomes in prostate cancer. Ongoing research and clinical trials will be critical in determining the best combination approaches and identifying the most effective strategies for targeting immunometabolism in this complex and challenging disease.

## 7. Conclusions

The TME plays a critical role in shaping the progression of prostate cancer, particularly through its influence on immunometabolism. Immunometabolism, the interplay between metabolic processes and immune cell function, has emerged as a crucial aspect of the immune response in cancer biology. In prostate cancer, the TME is characterized by nutrient deprivation, hypoxia and an accumulation of metabolic byproducts, all of which significantly impact both tumor cells and immune cells [122]. These factors contribute to an immunosuppressive environment that allows prostate cancer to evade immune surveillance and progress unchecked.

A key characteristic of prostate cancer is its capacity to reprogram metabolism to thrive in the challenging TME. Tumor cells shift from OXPHOS to glycolysis, even in the presence of oxygen, a process known as the Warburg effect [27,28]. This metabolic shift fuels rapid tumor progression by supplying both energy and the biosynthetic precursors needed for proliferation. Additionally, tumor cells heavily depend on other metabolic pathways, such as FAO and glutaminolysis, to endure nutrient deprivation and hypoxia [150,151]. These metabolic adaptations not only promote tumor progression but also alter the immune landscape by depriving immune cells of vital nutrients and producing immunosuppressive metabolites such as lactate and adenosine. Immune cells in the prostate cancer TME are significantly impacted by these metabolic changes. T-cells, crucial for antitumor immunity, are particularly affected by the TME’s metabolic constraints. The competition for glucose between tumor cells and T-cells, along with lactate accumulation, results in T-cell exhaustion and dysfunction. Similarly, macrophages in the TME are often polarized toward the M2 phenotype, which promotes tumor progression and suppresses immune responses, largely influenced by hypoxia and altered metabolic conditions [127]. MDSCs, dendritic cells and NK cells are also metabolically reprogrammed in ways that diminish their antitumor efficacy, further facilitating immune evasion [133,139,172].

While significant progress has been made in understanding the role of immunometabolism in prostate cancer, several gaps remain in the current knowledge. These gaps highlight the need for further research to fully elucidate the complex metabolic interactions within the TME and to develop novel therapeutic strategies that can exploit these metabolic vulnerabilities. One of the primary gaps in the current understanding of prostate cancer immunometabolism is the heterogeneity of the TME. The metabolic landscape of the TME can vary significantly between patients, and even within different regions of the same tumor, making it difficult to identify universal therapeutic targets. Future research should focus on characterizing the metabolic heterogeneity of the prostate cancer TME, with the goal of identifying patient-specific metabolic profiles that can guide personalized treatment strategies. Additionally, the dynamic nature of metabolism—shaped by changes in nutrient availability, oxygen levels and treatment pressures—presents a challenge for developing therapies that can effectively target metabolic pathways over time. Longitudinal studies that track metabolic changes in the TME throughout the course of prostate cancer treatment could provide valuable insights into how tumors adapt to metabolic therapies and inform the development of more effective interventions.

Another key area for future research is the development of metabolic inhibitors that selectively target tumor cells or immunosuppressive cells within the TME while sparing normal tissues. Current metabolic inhibitors often affect both cancer cells and healthy cells, leading to potential side effects and limiting their therapeutic utility [28,165]. Advances in drug development, particularly in the design of highly selective inhibitors, could help overcome these challenges. Moreover, identifying specific metabolic dependencies unique to prostate cancer cells or immunosuppressive cells like MDSCs and TAMs could open new avenues for therapeutic intervention. Exploring combination therapies that integrate metabolic inhibitors with existing immunotherapies is another promising direction for future research. Preclinical and clinical studies investigating these combination approaches are already underway, but further research is needed to optimize treatment protocols and identify the most effective combinations for prostate cancer.

Finally, there is growing interest in metabolic reprogramming as a therapeutic strategy. Rather than simply inhibiting metabolic pathways, reprogramming the metabolism of immune cells to enhance their function within the TME offers an innovative approach to cancer therapy. By boosting the metabolic fitness of T-cells and other immune cells, researchers aim to improve the immune response to prostate cancer, even in the face of the hostile TME [158]. Ongoing research into the molecular mechanisms underlying metabolic reprogramming in immune cells could reinvigorate the immune system and improve outcomes for prostate cancer patients.

In conclusion, the critical role of immunometabolism in prostate cancer progression underscores the need for continued research into the metabolic interactions within the TME. By exploring the metabolic vulnerabilities of both tumor cells and immune cells, researchers can develop novel therapeutic strategies that enhance immune responses, overcome the immunosuppressive nature of the TME, and improve treatment outcomes for prostate cancer patients.

## Figures and Tables

**Table 2:** Selective Clinical Trials of Immune Checkpoint Inhibitors in Prostate Cancer.

Drug	Target/Mechanism	Condition	Status (2025)	Phase	NCT Identifier
Ipilimumab	CTLA4	mCRPC	Completed	II	NCT02279862
Ipilimumab	CTLA4	mCRPC	Completed	III	NCT01057810
Ipilimumab	CTLA4	mCRPC	Completed	III	NCT00861614
Atezolizumab	PD-L1	mCRPC	Completed	I	NCT03024216
Atezolizumab	PD-L1	mCRPC	Active, not recruiting	III	NCT04446117
Durvalumab	PD-L1	mCRPC	Completed	II	NCT03204812
Avelumab	PD-L1	Neuroendocrine PCa	Completed	II	NCT03179410
Pembrolizumab	PD1	mCRPC	Recruiting	I/II	NCT02861573
Pembrolizumab	PD1	mCRPC	Completed	II	NCT03473925
Pembrolizumab	PD1	Hormone-sensitive PCa	Active, not recruiting	III	NCT04934722
Pembrolizumab	PD1	mCRPC	Active, not recruiting	III	NCT03834493
Pembrolizumab	PD1	mCRPC	Completed	III	NCT03834519
Nivolumab	PD1	mCRPC	Completed	II	NCT02601014
Nivolumab	PD1	mCRPC	Completed	III	NCT04100018
Ipilimumab + Nivolumab	CTLA4&PD1	mCRPC with CDK12 mutations	Completed	II	NCT03570619

## Data Availability

Not applicable.

## Abbreviations

The following abbreviations are used in this manuscript:

PCa	Prostate cancer
ADT	Prostate-specific antigen
CRPC	Castration-resistant prostate cancer
TME	Tumor microenvironment
OXPHOS	Oxidative phosphorylation
HIFs	Hypoxia-inducible factors
CAFs	Cancer-associated fibroblasts
ECM	Extracellular matrix
MDSCs	Myeloid-derived suppressor cells
Tregs	Regulatory T-cells
PD-L1	Programmed death-ligand 1
VEGF	Vascular endothelial growth factor
ROS	Reactive oxygen species
NO	Nitric oxide
NK cells	Natural killer cells
MHC	Major histocompatibility complex
TGF-β	Transforming growth factor beta
IL-10	Interleukin-10
PGE2	Prostaglandin E2
FAO	Fatty acid oxidation
TCA	Tricarboxylic acid
HK2	Hexokinase 2
PKM2	Pyruvate kinase M2
FASN	Fatty acid synthase
GLUTs	Glucose transporters
LDH	Lactate dehydrogenase
MCTs	Monocarboxylate transporters
ASCT2	Alanine-serine-cysteine transporter 2
GLS	Glutaminase
SQLE	Squalene epoxidase
LDL	Low-density lipoprotein
CAR-T	Chimeric Antigen Receptor T-cell
PSMA	Prostate-specific membrane antigen
2-DG	2-Deoxyglucose
